# Overview of 1q abnormalities in multiple myeloma: scientific opinions from Italian experts

**DOI:** 10.1007/s00277-025-06212-5

**Published:** 2025-02-13

**Authors:** Mattia D’Agostino, Marina Martello, Lorenzo De Paoli, Silvia Mangiacavalli, Daniele Derudas, Francesca Fazio, Anna Furlan, Carmine Liberatore, Giuseppe Mele, Roberto Mina, Roberto Ria, Elena Zamagni

**Affiliations:** 1 Division of Hematology, Department of Molecular Biotechnology and Health Sciences, AOU Città della Salute e della Scienza di Torino, University of Torino, Torino, Italy; 2 IRCCS Azienda Ospedaliero-Universitaria di Bologna, Istituto di Ematologia “Seràgnoli”, Bologna, Italy; 3 Dipartimento di Scienze Mediche e Chirurgiche, Università di Bologna, Bologna, Italy; 4 Hematology Unit, Ospedale Sant’Andrea di Vercelli, Vercelli, Italy; 5 Division of Hematology, IRCCS Fondazione Policlinico San Matteo di Pavia, Pavia, Italy; 6 Department of Hematology and Bone Marrow Transplant Center, Armando Businco Oncology Hospital, Cagliari, Italy; 7 Hematology, Department of Translational and Precision Medicine, Sapienza University of Rome – Azienda Policlinico Umberto I, Rome, Italy; 8 UOC di Ematologia Ca’ Foncello, AULSS2 Marca Trevigiana, Treviso, Italy; 9 Hematology Unit, Department of Oncology and Hematology, Ospedale Santo Spirito, Pescara, Italy; 10 UOC di Ematologia e Unità Trapianto di Midollo Osseo, Antonio Perrino Hospital, Brindisi, Italy; 11 Department of Precision and Regenerative Medicine and Ionian Area (DiMePRe-J), Internal Medicine “G. Baccelli”, University of Bari Aldo Moro Medical School, Bari, Italy; 12 Interdepartmental Centre for Research in Telemedicine, CITEL, University of Bari Aldo Moro, Bari, Italy

**Keywords:** Multiple myeloma, 1q abnormalities, Chromosome 1, +1q, Italy

## Abstract

Multiple myeloma (MM) is a haematological malignancy characterised by high genomic heterogeneity. One of the most common cytogenic abnormalities in MM is the gain of genetic material at the long arm (q) of chromosome 1 (+ 1q). While many mechanisms of resistance have been associated with + 1q alterations (e.g. CD38 downregulation, impairment of complement-dependent cytotoxicity, or induction of immunosuppression), the precise genetic or pathogenetic factors responsible for these alterations are still being investigated. Although interphase fluorescence in situ hybridisation (iFISH) is the gold standard for the detection of + 1q abnormalities used by the majority of diagnostic laboratories worldwide, there are no universally recognised cut-offs for + 1q positivity or a threshold for clinical meaningfulness. Because iFISH alone is insufficient to elucidate the extent of + 1q and other cytogenetic abnormalities in MM, sequencing-based methods could be adopted. The second revision of the international staging system for MM recently recognised + 1q as a high-risk feature. There is increasing evidence that + 1q has a prognostic value and influences the duration of remission, suggesting that patients with MM and + 1q may benefit from tailored therapy. This review comprehensively summarises the most recent biological evidence and clinical data on + 1q abnormalities in MM. However, given the heterogeneous data available, it remains difficult to draw firm conclusions. In clinical practice, +1q alterations should be evaluated along with other cytogenetic abnormalities and other biological and clinical characteristics of the disease. Ongoing and future studies will help the full understanding of the role of + 1q in MM.

## Introduction

Multiple myeloma (MM) is a haematological malignancy that is associated with the presence of monoclonal protein in blood and is characterised by the infiltration of the bone marrow by clonal terminally differentiated plasma cells [1]. The initiation of the pathogenetic process that leads to the emergence of MM occurs in the germinal centre. The mutation-bearing post-germinal centre B-cells migrate into the bone marrow, where they may give rise to monoclonal gammopathy of unknown significance, which in turn, may progress to smouldering MM, then to symptomatic MM, and eventually, to plasma cell leukaemia [1]. The disease is typically characterised by an important genomic complexity and heterogeneity at the precursor stages [2] responsible for diverse initiating events and clonal evolution leading to both inter-patient and intra-tumour genetic heterogeneity [3, 4]. Clonal evolution depends on a sequential acquisition of genetic alterations resulting in the formation of the initial clone and/or tumour subclones [3, 4]. A recent single-cell whole genome sequencing (WGS) study showed that genomic heterogeneity and clonal evolution is more complex than previously thought, and that gained genetic material can later be lost [5].

The two main pathogenetic groups are distinguished based on the presence of multiple trisomies (hyperdiploid MM) or translocations involving immunoglobulin gene loci (non-hyperdiploid MM) [3]. Amongst others, chromosome 1 abnormalities occur commonly in patients with MM [6]; abnormalities occur both in the short (p) and the long (q) arm of chromosome 1, and preferentially involve the loss of genetic material at 1p and its gain at 1q [7]. The − 1p deletions are observed in ~ 25% of patients with newly diagnosed MM (NDMM) and mainly affect 1p12 (genetic locus of the *FAM46C* gene) and 1p32.3 (genetic locus of the *CDKN2C* gene), with 1p12 and 1p32.3 deletions being present in 20% and 10% of patients with NDMM, respectively [7]. Abnormalities in 1p, such as biallelic deletion of 1p32, are independently associated with markedly poorer outcomes compared with a monoallelic loss [8, 9]. On the other hand, additional copies of 1q are common in MM and, similar to − 1p, are associated with poor prognosis [10].

**Fig. 1:**
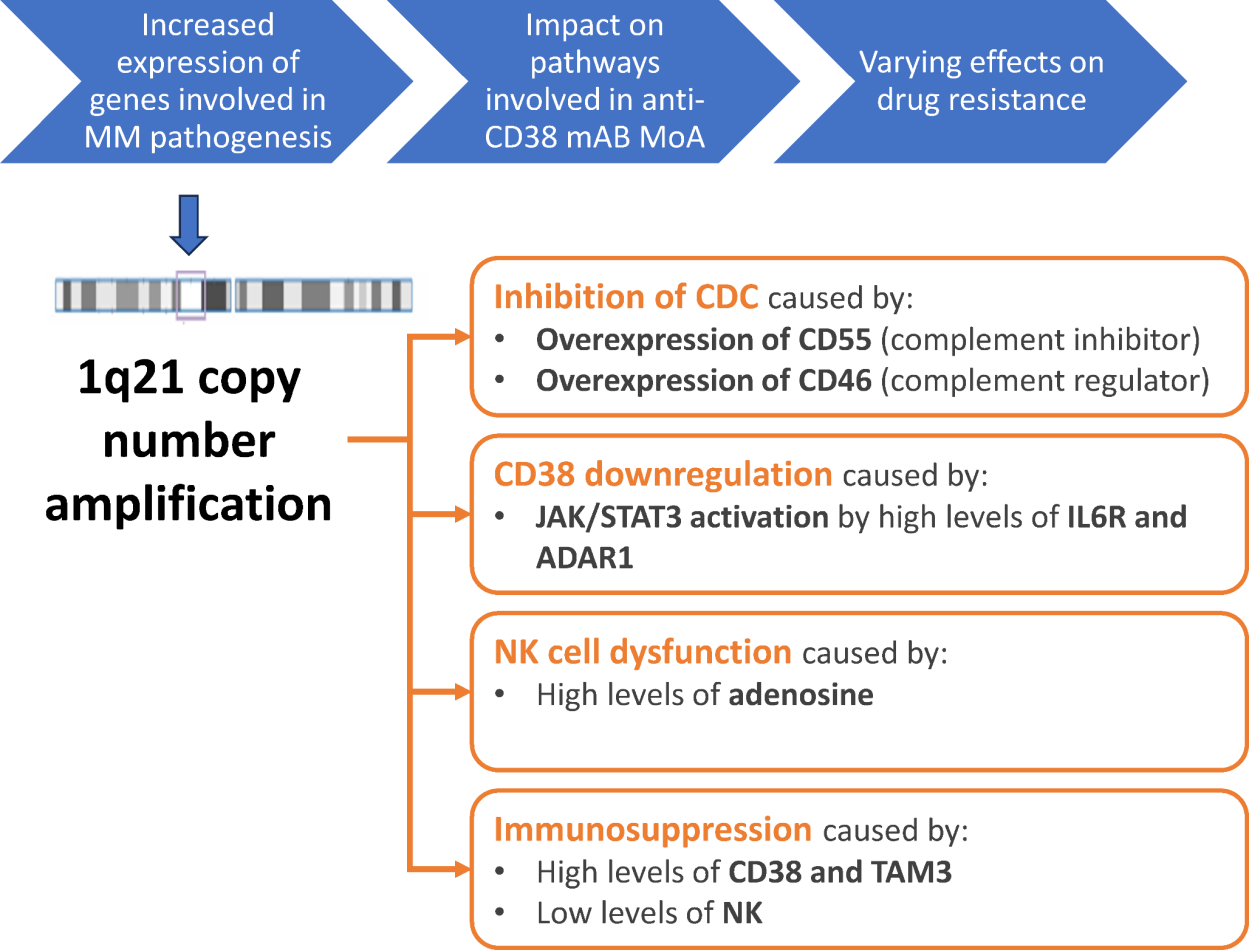
Pathogenic mechanisms activated through + 1q and involved in anti-CD38 resistance. +1q, gain of genetic material at the long arm of chromosome 1; ADAR, adenosine deaminase RNA specific; CD, cluster of differentiation; CDC, complement-dependent cytotoxicity; IL6R, interleukin-6 receptor; JAK, Janus kinase; mAb, monoclonal antibody; MoA, mechanism of action; MM, multiple myeloma; NK, natural killer; STAT, signal transducer and activator of transcription

Two types of 1q copy number alterations exist in MM and are referred to as gains or amplifications [11]. The term “gain(1q)” denotes acquisition of only one extra copy of chromosome 1q for a total of three copies, whereas “amp(1q)” corresponds to the presence of at least two extra copies of chromosome 1q for a total of four or more copies; “+1q” refers to any number of additional copies of any part of 1q [11]. Several copy number alterations that are harboured on 1q (particularly frequent in the 1q21 band [11]) may be relevant to the pathogenesis of MM (Fig. 1). As the 1q copy number increases, clinical outcomes become progressively worse [8, 12–14]. Copy number alterations involving 1q occur in ~ 40% of patients with NDMM [12]. Another study showed that the frequency of + 1q increases along the myeloma spectrum and is detected in ~ 17 − 23% of patients with monoclonal gammopathy of undetermined significance, 28 − 49% of patients with smouldering MM and/or NDMM, and up to 42–80% of patients with relapsed/refractory MM (RRMM) [15]. Moreover, the clonal size of + 1q cells within the tumour increases along the myeloma spectrum [16]. WGS analysis of samples from patients with RRMM or NDMM showed that the prevalence of + 1q increased from the levels found in NDMM to that found in lenalidomide- and pomalidomide-resistant disease, suggesting that the treatment acts as selective pressure that enriches drivers for + 1q abnormalities during the evolution of MM [17].

At present, there is no consensus on the region/gene within 1q that has the most relevant impact on outcomes in patients with MM. More work is needed to clearly understand the impact of + 1q alone or in combination with other cytogenetic abnormalities on disease prognosis. The aim of this review was to provide a comprehensive overview of the most recent biological evidence and clinical data on 1q abnormalities, including new scientific insights from Italian experts on MM.

## Mechanisms of + 1q acquisition and biological pathways involved

Gain(1q) and amp(1q) can be acquired through several molecular mechanisms [16]. The majority of the gain events are underlain by abnormal distribution of chromosome 1 copies during mitosis, whereas amplifications occur as a result of de-condensation of heterochromatin at 1q12, which may lead to jumping translocations, tandem duplications, or a combination of these events resulting in multiple copies of 1q21 seen in interphase fluorescence in situ hybridisation (iFISH) [16]. A gain(1q) can become an amp(1q) when extra copies (≥ 4) are acquired in a distinct moment of the disease evolution [18]. However, gain of genetic material is not always linked to gene overexpression and, consequently, to protein upregulation (e.g. cluster of differentiation [CD] markers) [19]. A recent analysis by Boyle et al. showed that the genetic region of chromosome 1 involved in copy number alterations had a variable length, ranging from 51 kb to 3.3Mb [20]. Using multiomic mapping, Boyle et al. identified nine regions of gain, seven of deletion, two of templated insertion and three of chromothripsis. The regions of gain tended to fall within the regions of active chromatin (i.e. regions with an open chromatin state, characterised by hypomethylation), resulting in a higher tendency of the genes included in these regions to be frequently overexpressed [20].

The CD38 cell surface molecule constitutes an important target of anti-MM treatment. The inhibition of complement-dependent cytotoxicity (CDC) is one of the proposed mechanisms of MM resistance to the anti-CD38 monoclonal antibody daratumumab [21, 22]. By contrast, another anti-CD38 monoclonal antibody, isatuximab, relies mainly on antibody-dependent (natural killer cells) cytotoxicity to destroy MM cells, implying that isatuximab treatment may be less affected by resistance mediated by *CD55* overexpression [23]. Interestingly, cells from extramedullary lesions from patients with MM display a lower expression of CD38. Extramedullary disease is a rare manifestation of MM associated with a poor prognosis. The finding of low expression of CD38 on cells from extramedullary lesions is frequently associated with + 1q and suggests the need for tailored treatment in these patients since extramedullary lesions may be resistant to anti-CD38 treatment [24]. Indeed, anti-CD38 antibodies also exert tumour destruction through immunomodulatory mechanisms such as induction of T-cell expansion [21].

Some genes have been implicated in the mechanisms of resistance to anti-CD38 antibodies relying on complement-mediated cytotoxicity [21]. Amongst the genes on 1q21, the overexpression of interleukin-6 receptor (IL6R) may enhance signalling from IL6, a cytokine with a known role in the pathogenesis of MM [25]. IL6R upregulation leads to CD38 downregulation through the activation of the Janus kinase (JAK)-signal transducer and activator of transcription (STAT) pathway, and involves an RNA editing protein called adenosine deaminase RNA-specific 1 (ADAR1), encoded by the *ADAR1* gene located in 1q21.3 [25, 26].

The gene that regulates CD55 (a complement system inhibitor otherwise known as complement-decay accelerating factor) is located outside of the 1q21 band [15]. CD55 regulates the complement system and inhibits the complement cascade [27]. CD55 expression increases on MM cells during disease progression, suggesting that its overexpression may be associated with a phenotype that is resistant to anti-CD38 antibodies, thereby leading to an inhibition of CDC [25, 28–30]. In fact, patients with CD55 overexpression may develop resistance to drugs that rely on CDC, such as daratumumab [22, 29].

Another gene of interest is the prune exopolyphosphatase 1 (*PRUNE1*) located in the 1q21.3 band. Overexpression of *PRUNE1* is often associated with the overexpression of the CDC28 protein kinase regulatory subunit 1B (*CKS1B*) gene [31]. *PRUNE1* promotes the proliferation and invasion of MM cells by stimulating purine metabolism, purine synthesis enzymes and mitochondrial functions, enhancing links between purinosomes and mitochondria. The downstream target of *PRUNE1* is CD73, which catalyses adenosine monophosphate breakdown to adenosine during extracellular metabolism and has been found to be overexpressed in many types of cancer; however, CD73 is inhibited by low pH in the MM bone marrow microenvironment [21, 31]. Accumulation of adenosine contributes to immunosuppression in patients with MM. Interestingly, CD38 is also involved in adenosine metabolism, and CD38 and CD73 inhibition may reduce adenosine production [21]. More research is needed to confirm the role of these genes in the pathogenesis of MM and/or to find other genes involved.

Outside of the 1q21 band, the PBX homeobox 1 (*PBX1*) gene, identified using a multiomic approach, is highly expressed in MM through amplification or through epigenetic mechanisms [32]. *PBX1* regulates *FOXM1*-dependent transcriptional program linked to proliferation of MM cells [32]. Amongst the genes located on other chromosomes, insulin-like growth factor-2 mRNA-binding protein-1 (*IGF2BP1*) located on chromosome 17q21.32 is highly expressed in patients with MM and concurrent + 1q alterations; high expression of *IGF2BP1* predicts a poor prognosis [33]. A 2023 study showed that *IGF2BP1* acts as a post-transcriptional enhancer of cell division cycle 5-like (CDC5L) protein in an N6-methyladenosine reader manner to promote the proliferation of MM cells with + 1q abnormalities; high *CDC5L* expression also predicts poorer prognoses in this patient population [34].

## Detection of 1q copy number alterations

Since + 1q alterations often coexist with other chromosomal defects, it is important to understand how abnormalities cluster so that personalised approaches to treatment can be determined [15, 35]. At present, there is no universally adopted cut-off for a 1q copy number alteration. Cut-offs may be suggested by guidelines or evaluated by individual laboratories using samples obtained from healthy volunteers; results are usually higher for copy number alterations than for translocations [36]. In addition, it is unclear whether laboratory determined cut-offs correspond to a clinically significant clone size [36]. Recent advances in automatic slide scoring have improved the statistics of failed tests, and has enabled the scoring of more nuclei and detection of unusual signal patterns; however, the future of + 1q detection will probably be fundamentally linked to the adoption of high throughput sequencing based methods [37, 38].

In the majority of laboratories, 1q abnormalities are detected using iFISH on CD138 + plasma cells that are isolated from bone marrow, which is still considered the gold standard of testing [15]. Although characteristic of an “old” and low-throughput technique, iFISH is a single-cell method that enables the analysis of prespecified abnormalities (usually up to 7–8 abnormalities due to material availability constraints), which limits the detection of additional abnormalities.

A universal adoption of next generation sequencing (NGS) would allow for simultaneous testing for multiple abnormalities to facilitate stratifying patients into subgroups according to their genomic abnormalities, including gain(1q), amp(1q), +1q and other alterations [37]. Given that there is currently no consensus on the most relevant region/gene on 1q, detailed DNA and RNA sequencing could help to match copy number alterations with expression data, pinpointing the most important regions involved [19]. Furthermore, proteomic studies would assist in the identification and verification of effective overexpression of a given CD marker or surface protein. WGS is a higher resolution method that can be used to identify all genetic abnormalities present in the cells from a patient with MM [17]. Another future development will involve the use of minimally invasive liquid biopsy to characterise genomic abnormalities in patients with MM to reduce the need for painful bone marrow biopsies [39].

## MM staging and + 1q in guidelines

Attempts to risk-stratify MM date back to Salmon-Durie staging in 1975 [40]. The international staging system (ISS) was first published in 2005 and was later revised in 2015 and 2022 [41–43]. The recognition of + 1q as a prognostic factor to be included in staging systems was introduced in the second revision of the ISS (R2-ISS) [41], in which patients were stratified into four groups based on the previously-recognised risk factors, plus new elements impacting on progression-free survival (PFS) and overall survival (OS), including del(17p), t(4;14), and + 1q [41]. However, the available scores do not consider high-risk clinical features, such as the presence and number of focal lesions, extramedullary MM, circulating malignant plasma cells, malignant plasma cells in bone marrow, plasma cell infiltration and the plasma cell proliferation index. A recent proposal for a scoring system includes the aforementioned clinical features and distinguishes between amp(1q) (considered a high-risk factor) and gain(1q) (considered a potentially high-risk factor) with a consequent indication that for the full understanding of the meaning of gain(1q), more data are needed [44]. In the latest version of the NCCN Clinical Practice Guidelines in Oncology (NCCN Guidelines^®^) on MM, both gain and amplification of + 1q21 are considered a high-risk factor, if not a sole abnormality, in patients with NDMM. In relapsed MM, the acquisition of + 1q and del(17p) or *TP53* mutation are factors considered as high-risk [45].

The available European Society for Medical Oncology (ESMO), National Comprehensive Cancer Network^®^ (NCCN^®^), International Myeloma Working Group (IMWG) and Stratification for Myeloma and Risk-Adapted Therapy (mSMART) guidelines are inconsistent regarding the definition of high-risk disease, testing and treatment recommendations [12, 45–47]. In particular, while IMWG and mSMART include + 1q in the definition of high-risk, and ESMO, NCCN and mSMART recommend including the + 1q probe in the iFISH panel [12, 45–47], none of them provide guidelines on how to treat patients with MM with + 1q alterations. The ESMO guidelines do not differentiate between the standard and high-risk NDMM when recommending treatment [12, 45–47]. In RRMM, therapeutic choices are based more on previous exposure to combinations of drugs with different mechanisms of action than on risk [46]. Also, the NCCN Guidelines^®^ are not risk-based [45]. The re-definition of risk and an IMWG consensus on 1q and high-risk is underway.

## Prognostic value of + 1q

The available data on the prognostic value of the presence of + 1q versus no 1q copy number alteration show high heterogeneity. In a retrospective analysis of 1376 patients with NDMM from the Mayo Clinic, +1q was associated with high tumour burden, advanced stage disease and the presence of high-risk translocations [48]. In addition, +1q was associated with decreased OS independent of other associated high-risk cytogenetic abnormalities, disease stage and age; however, no difference was observed in the overall response rate (ORR) or very good partial response (VGPR) between + 1q and no + 1q when treated with a proteasome inhibitor (PI), immunomodulatory imide drugs (IMiDs) or PIs + IMiDs [48]. In other studies, the impact of + 1q on clinical outcomes was observed in all stages of the myeloma continuum and when using multiple treatment modalities (from NDMM to RRMM) [15, 16, 28]. Kastritis et al. performed a prospective study in ~ 900 patients with NDMM to evaluate the prognostic value of + 1q21 and found that its presence was associated with more advanced disease, and inferior PFS and OS [49]. This was true especially in an ultra-high-risk group of patients with stage III disease and + 1q21, who were found to have very poor outcomes [49]. In the multivariate analysis to identify the features with the highest impact on OS and PFS that were used to build the additive R2-ISS score, +1q was a significant predictor for OS and PFS [41].

Available data concerning the prognostic value of gain(1q) versus amp(1q) are conflicting. In the series from the Mayo Clinic, there was no difference between gain(1q) and amp(1q) in terms of time to next treatment and OS [48]. In a meta-analysis of data from the NCRI Myeloma XI trial that included 1036 patients with NDMM with known iFISH + 1q status, a stratification by normal versus gain(1q) versus amp (+ 1q) was performed [50]. The presence of + 1q was associated with shorter PFS and OS compared with normal 1q copy number, but there was no significant difference between gain(1q) and amp(1q) [50]. On the other hand, in a retrospective study by Schmidt et al., amp(1q) was associated with worse PFS than gain(1q) (median PFS [mPFS]: 34 vs 56 months) [11]. A similar result was reported in the phase 2, randomised, open label FORTE trial, where 400 patients with NDMM were randomised 1:1:1 to carfilzomib + lenalidomide + dexamethasone (KRd)-autologous stem cell transplantation (d-ASCT), carfilzomib + cyclophosphamide + d-ASCT or 12 cycles of KRd [14]; mPFS was not reached in the normal 1q group, 54.9 months in the gain(1q) group and 21.2 months in the amp(1q) group. The presence of amp(1q) also predicted poor OS [10]. The adverse prognostic effect of + 1q occurring early or after the disease course in NDMM (even after the administration of first-line therapy) may be dependent on when the gain was acquired during the course of disease rather than the actual number of extra copies [13, 18].

## Outcomes in patients with + 1q

There are limited data from prospective clinical trials (ENDURANCE ECOG-ACRIN, FORTE, GRIFFIN, MAIA and GMMG-HD7) concerning the outcomes of patients with NDMM with gain(1q) or amp(1q) (Table 1). The ENDURANCE ECOG-ACRIN trial showed shorter PFS with + 1q (vs. those without + 1q) and with del1p (vs. those without del1p); when analysed by + 1q /del1p status within both treatment arms showed the same trends in PFS as the overall cohort [51]. Although a recent update of the GMMG-HD7 trial has shown significantly higher response rates and minimal residual disease (MRD) negativity rates in all treatment phases, subgroup analyses are awaited [52].

**Table 1:** Randomised clinical trials that analysed the outcomes in patients with NDMM and + 1q abnormalities

Trial name	*N*	FISH cut-off value	+ 1q prevalence	Regimen	PFS HR (95% CI) in + 1qsubgroups	Response
ENDURANCE ECOG-ACRIN [51] *Post-hoc analysis*	912	Not reported	34% +1q21 23% gain(1q) 8% amp(1q)	VRd vs. KRd	VRd HR vs. normal: gain(1q) 1.59, *P* = 0.012 HR amp(1q) 1.43, *P* = 0.186	VRd + 1q21: ORR: 79.9%, ≥VGPR: 65.1%
					KRd HR vs. normal: gain(1q) 1.41, *P* = 0.051 HR amp(1q) 2.37, *P* < 0.001	KRd + 1q21: ORR: 86.3%, ≥VGPR: 72.0%
FORTE [55] *Non-specified analysis type*	396	Not reported	32% gain(1q)	KRd-ASCT vs. KCd-ASCT vs. KRd12	**Gain(1q)** KRd-ASCT vs. KCd-ASCT: HR 0.35 *P* = 0.001 KRd-ASCT vs. KRd12: HR 0.45, *P* = 0.02 KRd12 vs. KCd-ASCT: HR 0.78, *P* = 0.38	Not reported
			12% amp(1q)		**Amp(1q)** KRd-ASCT vs. KCd-ASCT: HR 1.16, *P* = 0.72 KRd-ASCT vs. KRd12: HR 0.87, *P* = 0.73 KRd12 vs. KCd-ASCT: HR 1.34, *P* = 0.46	
GRIFFIN [56, 57] *Post-hoc analysis*	195	Not reported	29% +1q21	DVRd vs. VRd	**Gain/amp(1q)** DVRd vs. RVd: HR 0.42 (0.14; 1.27)	MRD – lasting ≥ 6 mo and ≥ 12 months (10^–5^) DVRd 41.2% vs. VRd 17.9%
					**Gain/amp(1q) + 1 HRCA** DVRd vs. VRd: HR 0.81 (0.15; 4.47)	DVRd 38.2% vs. VRd 14.3%
					**Isolated gain/amp(1q)** DVRd vs. VRd: HR 0.21 (0.04; 1.09)	MRD rate: DVRd vs. VRd OR 4.04 (95% CI 1.38; 11.81)
MAIA [63] *Post-hoc analysis*	737	Not reported	13% gain(1q) 20.4% amp(1q)	DRd vs. Rd	**Gain(1q) mPFS (months)** DRd vs. Rd: NR vs. 37.8 (HR 0.43 [0.24; 0.76])	MRD – lasting ≥ 12 months (10^–5^) **Gain(1q)** DRd vs. Rd: OR 4.91 (95% CI 1.31; 18.40)
					**Amp(1q) mPFS (months)** DRd vs. Rd: 40.0 vs. 26.1 (HR 0.81 [0.54; 1.21])	**Amp(1q)** DRd vs. Rd: OR 3.84 (95% CI 1.19; 12.38)
					**Isolated gain/amp(1q) mPFS (months)** DRd vs. Rd: 61.4 vs. 37.1 (HR 0.58 [0.40; 0.83])	**Isolated gain/amp(1q)** DRd vs. Rd: OR 4.53 (95% CI 1.87; 10.97)
GMMG-HD7 [94] *Subgroup analysis*	662	Cut-off levels for del(17p) and gain(1q21) were ≥ 10%	34.4% gain(1q21) (> 2 copies)	Isa-VRd vs. VRd	Primary endpoint: MRD	MRD- by NGF (10^− 5^) **Gain(1q21) (> 2 copies)**: 48.2% Isa-VRd vs. 35.6% VRd, OR 1.69

+ 1q, gain of genetic material at the long arm of chromosome 1; ASCT, autologous stem cell transplantation; CA, cytogenetic abnormality; CI, confidence interval; DRd, daratumumab + lenalidomide + dexamethasone; DoR, duration of response; DVRd, daratumumab + bortezomib + lenalidomide + dexamethasone; E, elotuzumab; HR, hazard ratio; HRCA, high-risk cytogenetic abnormalities; Isa, isatuximab; IMiD, immunomodulatory imide drug; KCd, carfilzomib + cyclophosphamide + dexamethasone; KRd, carfilzomib + lenalidomide + dexamethasone; KRd12, 12 cycles of KRd; m, median; MRD, minimal residual disease; NA, not available; NDMM, newly diagnosed multiple myeloma; NR, not reached; ORR, overall response rate; P, pomalidomide; PFS, progression-free survival; PI, proteasome inhibitor; Rd, lenalidomide + dexamethasone; TTP, time-to-progression; VRd, bortezomib + lenalidomide + dexamethasone; VGPR, very good partial response

PFS curves of data obtained in the FORTE trial were dependent on circulating plasma cells and MRD status; increasing levels of circulating plasma cells (above a cut-off) can constitute an easy-to-assess, robust and independent high-risk factor [53]. In addition, achieving MRD negativity reduced the negative prognostic impact of circulating plasma cells [53]. A 4-year analysis of PFS in patients with high-risk cytogenetic abnormalities, including gain(1q) or amp(1q), in FORTE showed worse survival in patients with amp(1q) than in patients with gain(1q) [54, 55].

An analysis of negative MRD (10^− 5^ sensitivity) was performed in patients with + 1q and revised cytogenetic risk in the GRIFFIN trial (daratumumab added to bortezomib + lenalidomide + dexamethasone [VRd]). A post hoc analysis showed MRD negativity lasting ≥ 12 months when daratumumab was added to VRd, suggesting a possible new standard of care for transplant-eligible NDMM [56, 57]. Facon and colleagues (2024) showed that in adults with NDMM who were ineligible to undergo transplantation, initial therapy with isatuximab added to VRd was more effective than VRd alone [58].

The OPTIMUM (MUKnine) phase 2 trial examined daratumumab + low-dose cyclophosphamide + VRd before and after ASCT in patients with ultra-high-risk (two or more high-risk cytogenetic abnormalities [HRCA]: t(4;14), t(14;16), t(14;20), +1q, − 1p, − 17p or high-risk SKY92 signature), NDMM or plasma cell leukaemia [59]. Although no specific data on + 1q were reported in this study, three or more HRCA, high-risk SKY92 signature or − 17p plus high-risk SKY92 increased the risk of early relapse despite intensified OPTIMUM treatment [60].

Patients with NDMM ineligible for ASCT in the MAIA trial were treated with daratumumab + lenalidomide + dexamethasone (DRd) or Rd alone [61]. Updated data of the trial after a median follow-up of 7.5 years has revealed that DRd is effective in maximizing survival in treatment-ineligible NDMM (median OS was 7.5 years) [62]. In a *post hoc* analysis of subgroups stratified according to cytogenetic risk, when treated with DRd, patients with isolated gain(1q21) demonstrated longer PFS than patients without HRCA who, in turn, showed longer PFS than those with an isolated amp(1q21) [63].

There are limited data on the prognosis and outcomes of patients with RRMM and + 1q abnormalities (Table 2). The ICARIA-MM, IKEMA and BOSTON trials performed stratification for + 1q in enrolled patients with RRMM, where the cut-offs for + 1q alterations were set at ≥30% (ICARIA-MM and IKEMA) [64–68] and ≥10% (BOSTON) [69].

**Table 2:** Randomised clinical trials that analysed the outcomes in patients with RRMM and + 1q

	Trial name	*N*	+ 1q prevalence	Regimen	PFS HR (95% CI) in +1q subgroups	Response, %
**Isatuximab**	ICARIA-MM [64, 65, 68] Phase III: *Post-hoc* analysis	307	41.7%	Isa-Pd vs. Pd	**Gain(1q) mPFS**: 9.5 vs. 3.8 m (0.40 [0.25; 0.63])	**Gain(1q)**: ORR, 53.9 vs. 17.3 (*P* < 0.001)
					**Amp(1q) mPFS**: 8.9 vs. 2.3 m (0.49 [0.24; 0.99])	
					**Isolated gain(1q) mPFS**: 11.2 vs. 4.6 m (0.50 [0.28; 0.88])	**Isolated gain(1q)**: ORR, 53.6 vs. 27.6 (*P* = 0.116)
					**Isolated amp(1q) mPFS**: 8.9 vs. 2.3 m (0.55 [0.21; 1.43])	**Isolated amp(1q)**: ORR 52.2 vs. 11.1 (*P* = 0.018)
	IKEMA [67] Phase III: +1q21 prespecified exploratory; gain(1q) and amp(1q), *post-hoc* analyses	302	42%	Isa-Kd vs. Kd	**Gain(1q)**: 0.50 (0.28; 0.90)	**Gain(1q)**: $\:\ge\:$VGPR, 73.3 vs. 51.9; CR, 41.3 vs. 25
					**Isolated gain(1q)**: 0.50 (0.27; 0.92)	**Isolated gain(1q)**: $\:\ge\:$VGPR, 80.9 vs. 51.6; CR, 46.8 vs. 22.6
					**Amp(1q)**: 0.73 (0.33; 1.63)	**Isolated amp(1q)**: $\:\ge\:$VGPR 65.6 vs. 40; CR, 34.4 vs. 26.7
**Elotuzumab**	ELOQUENT-2 [79] Phase III: Predefined subgroups	646	48%	ERd vs. Rd	**Gain(1q)**^**‡**^: 0.81 (0.61; 1.06)	Not reported
	ELOQUENT-3 [72] Phase II: Predefined subgroups	117	44%	EPd vs. Pd	**Gain(1q)**^**‡**^: 0.56 (0.29; 1.09)	
	SWOG-1211 [95]	100	47%	EVRd vs. VRd	**1q21**: HR 0.761 (0.459, 1.261)	
**Ixazomib**	TOURMALINE-MM1 [73] Phase II: Prespecified subgroups	722	NR	Ixa-Rd vs. Rd	**Isolated amp(1q) mPFS**: 15.4 vs. 11.3 m (0.78 [0.49; 1.24])	**Isolated amp(1q)**: ORR: 71 vs. 62; $\:\ge\:$VGPR 44 vs. 40, $\:\ge\:$CR 9 vs. 9
					**Expanded high-risk mPFS**^**†**^: 17.5 vs. 11.1 m (0.66 [0.47; 0.93])	**Expanded high-risk**: ORR: 75 vs. 65, $\:\ge\:$VGPR 45 vs. 33, $\:\ge\:$CR 10 vs. 6

*HR for experimental vs. control arms. ^†^Defined as those with high-risk CAs and/or 1q amplification. ^‡^Regardless of the presence of other high-risk CAs

+ 1q, gain/amplification of genetic material at the long arm of chromosome 1; CA, cytogenetic abnormality; CI, confidence interval; DoR, duration of response; ERd, elotuzumab + lenalidomide + dexamethasone; HR, hazard ratio; Ixa, ixazomib; Isa, isatuximab; IMiD, immunomodulatory imide drug; Kd, carfilzomib + dexamethasone; m, median; MRD, minimal residual disease; NA, not available; NR, not reached; ORR, overall response rate; Pd, pomalidomide + dexamethasone; PFS, progression-free survival; PI, proteasome inhibitor; Rd, lenalidomide + dexamethasone; RRMM, relapsed/refractory multiple myeloma; TTP, time-to-progression; Vd, bortezomib + dexamethasone, VRd, bortezomib + lenalidomide + dexamethasone; VGPR, very good partial response

A *post hoc* analysis of PFS, OS and response rate in the + 1q subpopulation of the ICARIA-MM trial revealed that the addition of isatuximab to pomalidomide [Pd] + dexamethasone improved PFS and OS versus Pd alone in patients with + 1q21 [64, 65, 68]. The PFS curves in the isatuximab-Pd arm appeared to be overlapping for patients with and without + 1q21, whilst patients in the Pd arm without + 1q21 had better PFS than those with + 1q21 [66].

The isatuximab-carfilzomib + dexamethasone (Kd) regimen was compared with Kd alone in the IKEMA study, which enrolled patients with RRMM who had received 1–3 previous lines of therapy and who were refractory or failed to achieve minimal response with prior anti-CD38 therapy [67]. The mPFS in patients with + 1q21 alterations was 25.8 months in the isatuximab-Kd arm and 16.2 months in the Kd arm [70]. In the isatuximab-Kd arm, patients with amp(1q21) had a worse mPFS than those with gain(1q21) (18.4 vs. 30.2 months); this difference was not apparent in patients treated with Kd alone [70]. PFS analyses in both ICARIA-MM and IKEMA showed that the presence of ultra-high-risk disease is an unmet need in the field [65, 67]. Importantly, the interpretation of these data is limited as there was no notable difference in patients treated with isatuximab-Pd with or without 1q abnormalities in ICARIA, but in IKEMA this difference was observed in the isatuximab-Kd arm [65, 67].

In the BOSTON study, the effects of selinexor were assessed in patients with MM who had exhibited high-risk (presence of del[17p], t[4;14], t[14;16] or ≥ 4 copies of amp1q21) cytogenetics (35.1%) and standard-risk cytogenetics (64.9%) [69]. The ORRs were 78.6% and 75.2%, respectively (OR 2.68; *p* = 0.004), suggestive that selinexor may be beneficial to patients with MM regardless of cytogenetic risk [69]. A real-world study from China confirmed the effectiveness and safety of selinexor-based regimens in patients with RRMM and HRCA, including + 1q21 [71].

In the ELOQUENT 3 trial, patients with RRMM refractory to lenalidomide and a PI were randomised to receive either elotuzumab-Pd or Pd alone [72]. Elotuzumab-Pd performed better than Pd alone and although patients with + 1q21 displayed a benefit when treated with elotuzumab-Pd, the presence of this abnormality was linked to a shorter duration of response [72].

The addition of ixazomib resulted in longer mPFS in patients with RRMM who were treated with ixazomib-Rd versus Rd alone in the TOURMALINE-MM1 trial [73]. A real-world Israeli study confirmed that ixazomib-based therapies were effective and safe in patients with RRMM, regardless of cytogenetic risk, resulting in a mPFS of 24 months, which is comparable with data obtained in clinical trials [74].

A recent systematic review assessed the current reporting trends of + 1q, the efficacy of existing regimens on + 1q and its prognostic implications in randomised controlled trials (RCTs) involving patients with MM [75]. Twenty-nine RCTs reported on + 1q, of which 10% defined cut-offs for + 1q, 14% separately reported survival data for gain(1q) and amp(1q) and ~ 80% considered + 1q alterations to be HRCA. The evidence for a statistically significant improvement of PFS in patients with MM and + 1q was shown in the experimental arm of Myeloma XI (lenalidomide maintenance arm vs. observation), BOSTON, IKEMA and ICARIA [65, 67, 69, 76]. Improvements in PFS in the + 1q cohort were also observed in the experimental arms of the Myeloma XI+, ELOQUENT-3 and HOVON-65/GMMG-HD4 trials; however, the 95% confidence intervals crossed 1 [72, 77, 78]. Despite the benefit of the applied interventions, all six studies showed worse OS and PFS in patients with + 1q alterations [65, 67, 69, 76, 79, 80], suggesting that further efforts are required to prolong these parameters. A systematic review has also noted considerable heterogeneity in the reporting of + 1q and that standardisation is needed [75].

In retrospective real-world studies, the impact of gain/amp(1q) was associated with significantly poorer outcomes at diagnosis and at relapse compared with standard-risk patients [15, 16, 28, 81]. Based on the available data, their presence can be considered a prognostic but not a predictive factor. Three retrospective analyses of daratumumab-based regimens showed conflicting results (Table 3) [28, 82, 83]. In one study, the presence of gain(1q) had an impact on mPFS (0.5 vs. 2.1 years) [28]. Instead, +1q21 had no effect on mPFS in the Parrondo et al. study, whereas, in the Barbieri et al. case series, amp(1q) had a dramatic impact on mPFS (3 months vs. not reached in patients without amp(1q)) [82, 83]. The latter case series reported on eight patients with RRMM and amp(1q) treated with a daratumumab-based triplet regimen (daratumumab-Rd in seven patients and daratumumab-Vd in one patient) [82]. In the Mohan et al. study, patients were first stratified into high- and low-risk molecular subgroups based on gene expression profile and further stratified by iFISH-detected gain(1q) or amp(1q) at the time of diagnosis or first visit [28]. The study population was heterogenous and comprised patients who had received up to 9 previous lines of therapy. Patients with + 1q21 (≥3 copies, cut-off 20%) had worse PFS and OS than those without + 1q21 when treated with daratumumab alone or in combination with an IMiD (most frequently, pomalidomide) [28]. The retrospective analysis from the Mayo Clinic included 278 patients with known + 1q21 status (mostly gain(1q), no cut-off reported) who had received 1–3 therapies with daratumumab (monotherapy or in combination) [48]. There were no reported differences in PFS among patients with or without + 1q21, patients with HCRA with or without + 1q21 and patients with standard risk with or without + 1q21 after treatment with daratumumab as monotherapy or in combination with Rd, Pd or Vd. The response rate at the level of ≥VGPR was 22.8% [83].

**Table 3:** Real world studies that analysed the outcomes in daratumumab-treated patients with MM

Study	*N*	Gain(1q) prevalence	Regimen	Outcomes	Response
Mohan et al. [28] Non-interventional	81	44.4%	D (mono or combination)	**Gain(1q) vs. without gain(1q)**: 0.5 vs. 2.1 years; *P* = 0.0004	Not reported
Parrondo et al. [83] Retrospective real-world analysis	278	20.5%	D (mono or combination)	**+ 1q21 vs. without + 1q21**: mPFS 24.5 vs. 23.5 months; *P* = 0.392	**+ 1q21 only**: ORR: 57.9%; ≥VGPR 22.8% No significant difference between patients with and without + 1q21 (*P* = 0.09)
Barbieri et al. [82] Non-interventional retrospective analysis	4	–	D-based triplet	**Amp(1q) vs. without amp(1q)**: 3.0 months vs. NR	Not reported
Lim et al. [81] Retrospective analysis of registry data	645	29.3%*	D (mono or in combination)	**+ 1q vs. without + 1q**: mPFS 8.2 vs. 18.4 months (HR 1.7, 95% CI 1.3–2.3; *P* < 0.001) **mOS in amp(1q) vs. gain(1q)**: 22.6 vs. 46.9 months; *P* = 0.035	**+ 1q vs. without + 1q**: no significant difference in ORR (65.9% vs. 71.6%; *P* = 0.32)

*iFISH data available in 451/654 patients

D, daratumumab; HR, hazard ratio; m, median; mono, monotherapy; NR, not reached; ORR, overall response rate; OS, overall survival; PFS, progression-free survival; VGPR, very good partial response

The Lim et al. study confirmed that + 1q21 often occurs in a cluster with other HRCA [81]. The negative impact of + 1q21 on PFS was not reversed by daratumumab and patients with amp(1q21) had even worse survival outcomes [81]. The predictive value of + 1q21 alone or together with other cytogenetic abnormalities was also evaluated in 737 patients with plasma cell neoplasms treated with current therapies in the real-world setting [84]. This study revealed that + 1q21 should be evaluated, irrespective of the R-ISS stage, together with other cytogenetic abnormalities and eligibility for ASCT. These factors are important because the predictive value of 1q21 gain or + 1q in MM depends on concurrent cytogenetic abnormalities and first-line treatment (no-ASCT with bortezomib + melphalan + prednisone, Vd, or Rd; ASCT with doxorubicin + Vd or cyclophosphamide + Vd or ASCT with bortezomib + thalidomide + low-dose dexamethasone or VRd) [84].

A retrospective study in China examined the impact of cytogenetic abnormalities in 328 consecutive patients with NDMM [85], where HRCA included del(17p), amp/gain(1q21), t(4;14) and t(14;16). The study confirmed the independent prognostic impact of + 1q21 [85]. Available real-world data confirm that it is useful to distinguish between gain and amplification and that + 1q should be evaluated in conjunction with other cytogenetic abnormalities. A retrospective study from the MD Anderson Cancer Center showed that patients with NDMM and + 1q, especially amp(1q), have inferior survival compared to standard-risk disease after upfront ASCT although better than those with other HRCA [13].

## Practical considerations in the clinical application of 1q21 alterations

Currently, the lack of consensus for standardized definitions and cut-offs for 1q21 gain/amplification limits the uniform application of 1q21 assessments in relation to timing of testing, test methods and instructions, interpretation of results and managing patients with + 1q abnormalities. In the past few years, several works have highlighted that 1q21 gain/amplifications detected both at diagnosis and relapse is important for risk stratification [86]. Indeed, 1q21 alterations are more frequent at relapse [87], clonal size of 1q21 positive cells is higher at relapse [16] and there is evidence that tiny, undetectable subclonal 1q21 positive cells can guide the determination of relapse [88]. Current European Myeloma Network (EMN) guidelines include 1q21 alterations in the panel of tests to ascertain risk alterations [36]; in usual clinical practice, the entire panel is tested whenever iFISH is performed. Thus, we suggest that 1q21 alterations should be assessed routinely by iFISH both at diagnosis and at relapse.

While conventional cytogenetic testing is no longer recommended in MM and has long ceased to be used in clinical practice, the implementation of a customized NGS targeted panel (e.g. a unique molecular assay [UMA] panel [89]) can provide additional information as it is able to detect copy number alterations at the whole-genome level, structural variants, and mutations on selected genes in a single assay [89]. Although NGS techniques are not yet widespread, they represent a technological advancement, to which a number of laboratories are adapting in order to confirm the diagnosis of various diseases, including MM [37].

Regarding cut-offs, the most widely used to define positivity for 1q21 abnormalities are those from the EMN guidelines, where positivity corresponds to detection of these abnormalities in ≥ 20% of analysed cells [36]. In a report on the number of 1q copies, a 20% cutoff is also applicable to cells with at least 4 copies of 1q to define Amp(1q) [14]. However, since the frequency of 1q21 gain/amplification might increase as the disease progresses, HCPs should be alerted when 1q21 alterations are present in subclones at diagnosis (< 20% of cells) [28, 32]. This evidence strongly justifies the re-assessment of 1q21 alterations at relapse. On the other hand, a 10% cutoff is the limit that is applied to normal bone marrow using the mean ± standard deviation of three abnormal signals detected in the bone marrow plasma cells of healthy donors [14]. As such, we recommend that when iFISH is used, a cut off < 10% is not considered a positive result for 1q21 alterations.

There is much debate on whether 1q positivity should be considered as a standalone high-risk factor or high-risk only when associated with other chromosomal abnormalities. Several reports indicate that + 1q is an independent predictor of PFS and OS in multivariate analysis [41, 50]. However, there is a clear “double hit” effect when + 1q is associated with other high-risk features, where the prognosis is typically worse [17]. Notably in the FORTE trial, at least 4 copies of 1q (amp(1q)) without additional HRCA had a similar survival outcome compared with gain(1q) with additional HRCA (mPFS: 35.2 months vs. 35.1 months). These data suggest that amp(1q), even as a standalone chromosomal abnormality, should be considered high risk. Indeed, the worst outcome was detected in patients with amp(1q) with additional HRCA (mPFS: 10.9 months), suggesting that there is an additive effect of concomitant HRCA in the context of amp(1q) [14]. As with other high-risk markers, clinicians should interpret 1q positivity as a purely prognostic marker. With the present evidence, and as stated in the ESMO guidelines, no prognostic factor or staging system should be used to define a risk-adapted strategy [46]. However, single arm phase II trials adopting intense treatment strategies in high-risk patients with MM produced better outcomes than historical controls [59, 90, 91] and randomised trials investigating risk-adapted treatments are ongoing (e.g. the RADAR trial [EudraCT: 2019-001258-25] [92]).

Regardless of the type of treatment, close disease monitoring is essential in patients with MM. Obtaining and maintaining MRD negativity should be the main treatment goal [93].

## Conclusion

Cytogenetic abnormalities are among the most powerful predictors of patient outcomes. Although it is difficult to draw firm conclusions about the role of + 1q in MM given the heterogenous data available in the literature, evaluation of + 1q, together with other cytogenetic abnormalities, should be mandatory at clinical trial enrolment and increasingly requested in clinical practice, along with the assessment of other biological and clinical characteristics of the disease. Moreover, determination of specific genomic alterations that may suggest the overexpression of specific surface markers (e.g. CD38, CD55, IL6R) has a crucial role to better define the risk profile. This is preferably accomplished by the adoption of sensitive high-throughput methods like genome sequencing. Identifying patients with + 1q alterations will become increasingly important as the presence of this cytogenetic abnormality may guide the choice of treatment in future. Ongoing and future studies will help the full understanding of the role of + 1q alterations in MM.

## Data Availability

No datasets were generated or analysed during the current study.

## Abbreviations

+ 1q: Gain of genetic material at the long arm of chromosome 1
ADAR: Adenosine deaminase RNA specific
ASCT: Autologous stem cell transplantation
CD: Cluster of differentiation
CDC: Complement-dependent cytotoxicity
CDC5L: Cell division cycle 5 like
EMN: European Myeloma Network
ESMO: European Society for Medical Oncology
DRd: Daratumumab + lenalidomide + dexamethasone
HRCA: High-risk cytogenetic abnormalities
iFISH: Interphase fluorescence in situ hybridisation
IGF2BP1: Insulin-like growth factor-2 mRNA binding protein 1
IL6R: Interleukin-6 receptor
IMiDs: Immunomodulatory imide drugs
IMWG: International Myeloma Working Group
JAK: Janus kinase
Kd: Carfilzomib + dexamethasone
KRd: Carfilzomib + lenalidomide + dexamethasone
mSMART: Stratification for Myeloma and Risk-Adapted Therapy
MM: Multiple myeloma
mPFS: Median progression-free survival
MRD: Minimal residual disease
NCCN: National Comprehensive Cancer Network^®^ (NCCN^®^)
NDMM: Newly diagnosed multiple myeloma
NGS: Next generation sequencing
ORR: Overall response rate
OS: Overall survival
Pd: Pomalidomide + dexamethasone
PFS: Progression-free survival
PI: Proteasome inhibitor
q: Long arm
Rd: Lenalidomide + dexamethasone
RCT: Randomised controlled trial
R2-ISS: Second revision of the international staging system
RRMM: Relapsed/refractory multiple myeloma
STAT: Signal transducer and activator of transcription
Vd: Bortezomib + dexamethasone
UMA: Unique molecular assay
VRd: Bortezomib + lenalidomide + dexamethasone
VGPR: Very good partial response
WGS: Whole genome sequencing
